# COVID-19 Breakthrough Infections among Patients Aged ≥65 Years in Serbia: Morbidity and Mortality Overview

**DOI:** 10.3390/vaccines10111818

**Published:** 2022-10-28

**Authors:** Monika P. Bajči, Dajana F. Lendak, Mioljub Ristić, Maja M. Drljača, Snežana Brkić, Vesna Turkulov, Vladimir Petrović

**Affiliations:** 1 Department of Infectious Diseases, Faculty of Medicine, University of Novi Sad, Hajduk Veljkova 3, 21112 Novi Sad, Serbia; 2 COVID Hospital, University Clinical Center of Vojvodina, Mišeluk bb, 21131 Petrovaradin, Serbia; 3 Clinic for Infectious Diseases, University Clinical Center of Vojvodina, Hajduk Veljkova 1, 21112 Novi Sad, Serbia; 4 Department of Epidemiology, Faculty of Medicine, University of Novi Sad, Hajduk Veljkova 3, 21112 Novi Sad, Serbia; 5 Centre for Disease Control and Prevention, Institute of Public Health of Vojvodina, Futoška 121, 21000 Novi Sad, Serbia

**Keywords:** COVID-19, breakthrough infection, older adults, geriatric medicine

## Abstract

**Highlights:**

**What are the main findings?**

1. Case fatality ratio decrease and reduction of severe and critical forms have been noted in fully vaccinated older individuals.
2. Increasing age is still an independent factor for disease severity and poorer outcome of COVID-19 unrelated to individual vaccine status.

**What is the implication of the main finding?**

3. Providing evidence on health benefits for vaccinated older adults in terms of reducing disease severity and poor outcome.
4. Focus can be shifted to investment in and implementation of preventive forms of medicine, such as vaccination.

**Abstract:**

BACKGROUND: Vaccines against severe acute respiratory syndrome coronavirus 2 have shown effectiveness in the prevention of COVID-19. Breakthrough infections occur, and age has been shown to be one of the dominant risk factors for poorer outcome. This research focuses on characteristics of breakthrough infections in older adults. METHODS: This retrospective study was conducted for four months (March–June 2021) in the autonomous province of Vojvodina in Serbia on 11,372 patients using reverse-transcription polymerase chain reaction or antigen-detection rapid diagnostic tests verifying COVID-19 in those aged ≥65 years. Demographics, comorbidities, disease severity, and final outcomes were evaluated in fully vaccinated compared to unvaccinated individuals. Individuals were divided into younger-old (65–74 years) and older-old (≥75 years) age groups and differences between those groups were further evaluated. Binary logistic regression was performed to identify independent predictors of poor outcome. RESULTS: By the end of the research, 51.3% of the population of APV 65–74 years, as well as 46.2% of those older than 74 years, were vaccinated. From the acquired sample, 17.4% had breakthrough infection. Asymptomatic forms were higher in both age groups of vaccinated vs. unvaccinated (3.9%—younger-old, 6.3%—older-old vs. 2.9%—younger-old, 3.9%—older-old). The same results were registered with mild symptoms (82.1%—younger-old, 68.1%—older-old vs. 76.3%—younger-old, 57.5%—older-old) (*p* < 0.001). The case fatality ratio of the vaccinated population was smaller than the unvaccinated population in both groups (3.1% vs. 7.9%—younger-old; 11.4% vs. 22.5%—older-old) (*p* < 0.001). The odds ratio for poor outcome in unvaccinated individuals was 2.3 (95% confidence interval, *p* < 0.001) for the total sample. CONCLUSIONS: An increase in asymptomatic and mild forms, as well as decrease in severe or critical forms and poor outcomes, were noted in the vaccinated population. Choosing to avoid vaccination against SARS-CoV-2 may increase the chance of poor outcome in older individuals.

## 1. Introduction

Since December 2019, the world has been facing a global health crisis. It emerged as an increased incidence of atypical pneumonia of unknown origin, which rapidly spread throughout the globe. The aetiology of the pneumonia was identified and named severe acute respiratory syndrome coronavirus 2 (SARS-CoV-2), and the disease was named COVID-19 [1].

Clinical presentation included upper and lower respiratory tract infections, though there are records of the disease manifesting itself through complications such as acute respiratory distress syndrome, disseminated intravascular coagulation, and multiple organ dysfunction syndrome [2,3]. The term ‘cytokine storm’ explained part of the pathophysiological process, making COVID-19 not only an infectious but also an immunologic disease according to its mechanism of action [4]. With the processes of involution of immune function that come with increasing age, also known as immunosenescence, older adults are at a higher risk of infections [5].

Vaccines against COVID-19 are effective in decreasing incidence and mortality rates, as well as in reducing the case fatality ratio caused by SARS-CoV-2 [6]. Lopez et al. suggest that Pfizer-BioNTech and Oxford-AstraZeneca vaccines reduced symptomatic COVID-19 among people aged 70 years and older [7]. In Serbia, the vaccination campaign started on 24 December 2020. The Serbian Medicines Agency approved the use of four vaccines in the fight against COVID-19: Pfizer-BioNTech BNT162b2, Gamaleya Research Institute Gam-COVID-Vac, Oxford/AstraZeneca ChAdOx1 nCoV-19 AZD1222 and Sinopharm BBIBP-CorV [8].

A recently conducted cross-sectional study showed that the strongest antibody response was measured in the group vaccinated by BNT162b2, followed by Gam-COVID-Vac and BBIBP-CorV. The study also confirmed that BNT162b2 and Gam-COVID-Vac stimulated more antibody production than in patients who recovered from COVID-19 [9]. However, vaccines do not offer 100% protection and breakthrough infections can still occur. Their clinical presentation mostly includes mild and asymptomatic forms [10]. Older people represent more liable targets for breakthrough infections due to impaired vaccine response coupled with the decreased vital capacity of organs and the occurrence of comorbidities [11]. A nationwide study done in Hungary showed that the effectiveness of the vaccine against SARS-CoV-2 infection decreases and the death rate from COVID 19 increases with an increase in the age of the population studied [12]. More precise results deriving from hospitalized patients showed that the totally vaccinated group was mainly composed of older males with several comorbidities [13].

The first aim of this study was to display the differences in clinical presentations and outcomes of COVID-19 infections between unvaccinated and fully vaccinated older individuals. The second aim was to predict how vaccination status and comorbidities affect the total disease outcome. Our hypothesis was that clinical presentation and disease outcome are more favourable in the fully vaccinated elderly population.

## 2. Methods

### 2.1. Study Population

For this retrospective study, information was collected from the whole population of the autonomous province of Vojvodina (APV) during a four-month period (1 March 2021 to 30 June 2021). We used data from the APV and Serbia COVID-19 surveillance registry, which contains sociodemographic and epidemiological characteristics of all COVID-19 laboratory-confirmed cases. We adopted the definition of breakthrough infection as a case of confirmed (by reverse-transcription polymerase chain reaction (RT-PCR) or rapid antigen-detection diagnostic test (STANDARD Q COVID-19 Ag test) [14]) SARS-CoV-2 infection in fully vaccinated (two doses administered) individuals where at least 14 days passed between receiving the second dose of SARS-CoV-2 vaccine and prior to the occurrence of the first symptoms [8,15].

Ethics approval was obtained prior to conducting this study from the ethics committee of the Clinical Centre of Vojvodina (00–150, date of approval 13 August 2021). Informed consent was waived due to the study’s observational nature and the anonymity of data used for the analysis.

Only participants aged ≥65 years were considered. Demographics, the clinical presentation of COVID-19 along with information about comorbidities, date of the symptoms’ onset, the severity and outcome of the disease (recovery/lethal), and vaccination status (yes—two doses of vaccine/no) with the date(s) of their administration among vaccinated patients were included. Recovered patients were in no need of hospitalization or had been discharged from with no need for rehospitalization in the 8 weeks following noted SARS-CoV-2 positivity. Patients with lethal outcomes also had COVID-related poor outcomes in the first 8 weeks from infection. All vaccinated patients received the same type of vaccine for their first and second dose and followed the recommended time schedule between doses (3 weeks between the first and second dose for Pfizer-BioNTech BNT162b2, Gamaleya Research Institute Gam-COVID-Vac, and Sinopharm BBIBP-CorV, and 12 weeks between two doses for Oxford/AstraZeneca ChAdOx1 nCoV-19 AZD1222 vaccine). Demographic characteristics included the age and gender of the participants. Comorbidities (hypertension, cardiovascular disease, chronic obstructive pulmonary disease, diabetes mellitus, obesity, malignancy, and others; ischemic stroke, arrhythmias, renal insufficiency, psychiatric disorders, neurologic disabilities, heart valve disease, peripheral vascular disease) were considered as dichotomous categorical variables.

Enrolled patients were divided into groups according to their age and vaccination status. The total sample was divided into two age categories: younger-old and older-old adults. The younger-old category contained individuals aged 65 to 74 years, and the older-old consisted of participants aged 75 years and older [16]. Vaccination status divided individuals into vaccinated and unvaccinated. Vaccinated individuals were those who had first symptoms of the disease or asymptomatic laboratory confirmation 14 or more days after the second dose. Falling into the unvaccinated category implied that the individual was not vaccinated at all, was vaccinated with the first dose, or was vaccinated with the second dose, but the first symptoms of the disease or asymptomatic laboratory confirmation occurred less than 14 days after the administration of the second dose, regardless of the type of SARS-CoV-2 vaccine.

Clinical presentation of COVID-19 was defined through four categories: asymptomatic, mild, severe, and critical. All participants who had only laboratory confirmation of SARS-CoV-2 infection obtained by either RT-PCR or standard Q COVID-19 Ag test without any signs/symptoms of COVID-19 were considered asymptomatic. Testing of asymptomatic individuals was indicated in cases of close contact with an individual who was confirmed to have COVID-19, in shared living facilities, prior to traveling, surgical procedures, receiving immunosuppressive therapy, or in need of hospitalization for indications other than COVID-19. Mild disease forms included all individuals who experienced clinical signs/symptoms (including radiograph-verified pneumonia) of COVID-19 without the need for oxygen support. Severe and critical forms needed hospitalization. Severe forms needed oxygen support via non-invasive methods (oxygen mask, nasal cannula, and continuous positive air pressure machine). Critical forms were supported in intensive care units (ICU) with invasive mechanical ventilation techniques.

### 2.2. Statistical Analysis

Statistical analysis was performed using IBM SPSS version 23.0 (IBM, Chicago, IL, USA). Categorical variables were compared using a Chi-square test and are presented as numbers and percentages. Continuous variables are presented as arithmetic means and standard deviations and were tested via *t*-test.

Binary logistic regression was performed to identify independent predictors of poor outcomes of COVID-19. The strongly correlated variables were excluded to avoid multicollinearity. Based on the *p*-value of the results obtained in the univariate analysis, the variable ‘cardiovascular disease’ was excluded, because of multicollinearity with hypertension. A value of *p* < 0.05 indicated statistical significance, and the confidence interval was 95%.

## 3. Results

During the study period (1 March to 30 June 2021), 11,372 people aged 65 years and older were tested positive for SARS-CoV-2 in the APV. By the end of the research, 51.3% of the population of the APV aged 65–74 years (younger-old) and 46.2% of those older than 74 years (older-old) were vaccinated with two doses of one of the four SARS-CoV-2 vaccines. The total number of breakthrough cases was 1975—17.4% of the tested population. Of all the registered breakthrough cases, 1272 were aged 65 to 74 years (64.4%) and 703 cases were 75 years and older (35.6%) (Table 1). The total population of APV incidence of COVID-19 for younger-old participants was 3840 per 100,000 individuals (incidence rate (IR) of breakthrough among vaccinated younger-old was 1290 and for unvaccinated 6527). For the older-old age group, IR was 2282 (IR for breakthrough among vaccinated older-old participants was 850 and for unvaccinated 3511). Gender structure revealed that 5103 (44.9%) of the total sample were men, while in younger-old patients they took up 46%, and in older-old ones 42.8% of the group population (Table 1).

In older-old participants, hypertension and cardiovascular diseases were more common in the unvaccinated population than in the vaccinated (hypertension: 41.1% vs. 36.7%, *p* = 0.037, cardiovascular diseases: 11.0% vs. 7.1%, *p* = 0.002), but obesity was more frequent in vaccinated individuals than in unvaccinated ones (2.1% vs. 0.9%, *p* = 0.003) (Table 1).

**Figure 1:**
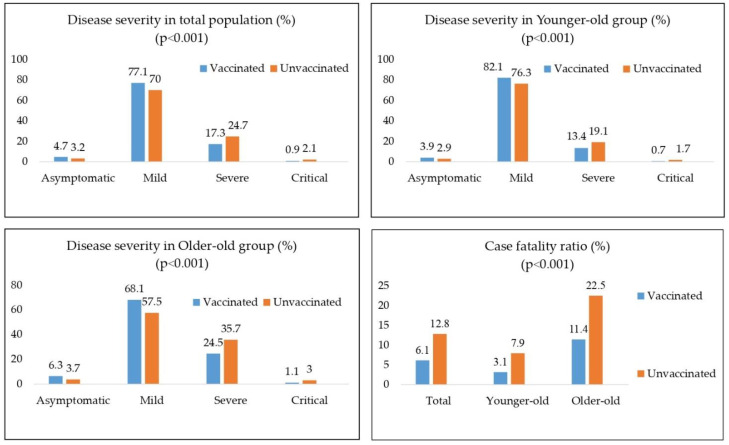
Case fatality ratio and differences in disease severity in total, Younger-old and Older-old age group regarding vaccination status.

Regarding the severity of COVID-19, asymptomatic forms of the disease were higher in both age groups in vaccinated vs. unvaccinated (3.9%—younger-old, 6.3%—older-old vs. 2.9%—younger-old, 3.9%—older-old). The same pattern continued in the group with mild symptoms (82.1%—younger-old, 68.1%—older-old vs. 76.3%—younger-old, 57.5%—older-old). This increase in asymptomatic and mild forms was followed by a registered decrease in severe and critical forms in both vaccinated groups (Figure 1).

In terms of the outcome of the disease, similar results were found: case fatality ratio of the vaccinated population was lower than the unvaccinated population in both age groups (3.1% vs. 7.9%—younger-old; 11.4% vs. 22.5%—older-old) (*p* < 0.001) (Figure 1).

In Table 2, an analysis of the lethal outcomes of COVID-19 in the whole examined population (older than 65) as well as in divided groups (younger-old aged 65–74, and older-old aged 75 and more) is shown. For the whole population, the mean age of the deceased was 77 years vs. individuals who survived, who were 72 years old on average (*p* < 0.001). For age ≥75 years, the difference was 82 years vs. 80 years (*p* = 0.001) (Table 2). Comparing comorbidity structure with disease outcome in the whole population showed that every comorbidity, except obesity, was in greater numbers present in those with the poorer outcome than those who recovered (*p* < 0.001) (Table 2).

After that, binary logistic regression analysis was performed, with an intention to extract variables that are independent predictors of the lethal outcome in the whole examined population (older than 65—Table 3), as well as in younger-old (Table 4) and older-old group (Table 5). For the whole examined population, the analysis showed that vaccination status (unvaccinated group), age, hypertension, chronic obstructive pulmonary disease, diabetes mellitus, obesity, and malignancy were all independent predictors of lethal outcome in COVID-19 (*p* < 0.001). The odds ratio (OR) for the unvaccinated was 2.3 (95% confidence interval (CI) 1.9–2.9). Age showed an OR of 1.10 (95% CI 1.09–1.11). The first three comorbidities that showed notable individual impact on COVID-19 mortality were malignancy with OR = 6.3 (95% CI 4.2–9.8), chronic obstructive pulmonary disease OR = 5.5 (95% CI 3.9–7.9), and diabetes mellitus OR = 3.6 (95% CI 2.9–4.5) (Table 3).

In the younger-old group, the unvaccinated individuals had 2.8 times more chance of lethal outcome (OR = 2.8; 95% CI 2.0–3.9) (Table 4), while in the older-old group, the OR for lethal outcome in unvaccinated was 2.1 (OR = 2.1; 95% CI 1.6–2.7) (Table 5).

## 4. Discussion

Our study showed three significant results:

- Lower incidence rate of COVID-19 infection in vaccinated people in the whole >65 years old population
- Among SARS-CoV-2-positive people, we noticed a significantly greater percentage of asymptomatic and mild forms of the diseases in the vaccinated group than in the unvaccinated group
- Vaccination status is an independent predictor of the lethal outcome, with odds ratio of 2.3, as expected in the hypothesis.

Although a significantly lower incidence occurred in the vaccinated compared to the unvaccinated group (younger-old group 1290/100,000 vs. 6527/100,000; older-old group 850/100,000 vs. 3511/100,000), we have documented a noteworthy number of breakthrough infections in our study. There are multiple explanations that can give possible answers to why breakthrough infections occur. Considering the product itself, different vaccine platforms showed a discrepancy in the effectiveness of the vaccines [9,17,18,19]. Some of the platforms are more demanding than others when it comes to the conditions of the distribution and storage. Disregard for one of the steps in the supply chain could lead to a decrease in the effectiveness of a vaccine. Another potential explanation for the occurrence of breakthrough infection could be found in the ability of the virus to mutate, thereby avoiding the reaction of the immune system of a vaccinated host, which also decreases the effectiveness of a vaccine and increases the possibility of breakthrough infection [20]. The reaction of the individual itself could also be considered important for the outcome. Wei et al. identified a ‘low responder’ group that included people aged >75 years, males, and people with chronic conditions, which were all immunosuppressed [21]. Current knowledge about COVID-19 is that age is an independent risk factor for the infection. Moreover, it often results in admission to an ICU as well as lethal outcome [22]. Fulop et al. showed that optimal immune response decreases at the age of approximately 60 years [23]. Similarly, an age-related decline in immune response was noted for influenza and pneumococcal vaccines [24], which could explain why the vaccine failure increases with aging. All these indications could lead to the unfolding of mechanisms responsible for breakthrough infections. Responsible and punctual decisions should be implemented on various fronts in an effort of sustaining their occurrence.

The aim of this research was to form a more accurate description of SARS-CoV-2 in infected older adults. The statistical analysis of comorbidities showed that vaccinated adults aged ≥75 were in fact healthier than the unvaccinated comparison group, with unvaccinated more often suffering from hypertension and cardiovascular diseases. The first possible explanation for this phenomenon could lie in the fact that patients with comorbidities can manifest SARS-CoV-2 positivity earlier thanks to the exacerbation of the underlying chronic conditions, while the comorbidity-free group could receive a proper diagnosis of COVID-19 in a prolonged time [25]. Surveys have confirmed that the delay between the onset of symptoms and diagnosis may increase mortality rates [26]. The second explanation for this phenomenon could be found in human behaviour after vaccination. Previously healthy and fully vaccinated individuals may show a tendency for premature unmasking and possible risky exposure to crowds and mass gatherings. The third explanation lies in the healthcare system itself. General practitioners’ hesitance about vaccination against COVID-19 could affect patients’ decisions not to get vaccinated, especially in the case of patients with comorbidities, which leaves them in the unvaccinated group when the infection occurs [27,28].

The study of Vokó et al. suggested that the efficacy of the Sinopharm vaccine is diminished in adults aged 65 years [12]. The fact that in Serbia, the same age category of individuals was immunized mostly with Sinopharm (68.8% of all vaccinated individuals in these age groups), explains the percentage of breakthroughs [8]. A more specific analysis of the early effectiveness of the four COVID-19 vaccines in the period between January and April 2021 in Vojvodina showed that among the participants vaccinated with the first dose, 86% received BBIBP-CorV, 7% BNT162b2, 5.1% Gam-COVID-Vac and 1.9% ChAdOx1 nCoV-19 vaccine [29]. These results could implicate why previously healthy older adults suffered breakthrough infections.

The most significant result of this research is the effect of the vaccination process on the severity of the disease and its final outcome. In both age groups, disease severity shifted from critical and severe forms to mild and asymptomatic ones. Results of a study published by other authors showed that breakthrough infections manifested themselves in most cases as mild or asymptomatic, with a shorter duration of the disease in the adult population [30]. As expected, patients younger than 75 years showed better results because the physiological course of aging with reduced lung reserve, and diminished airway clearance makes older individuals more prone to the disease [31]. However, the process of vaccination has made an impact on stimulating the immune system, which later made a difference in the total progression of the disease in both age groups.

The final outcome showed that vaccinated old adults have a reduced case fatality ratio of half compared with unvaccinated ones in their age group. Binary regression analysis showed that ‘unvaccinated’ status presents an independent predictor of poor outcome, increasing the chances of unwanted outcome by 2.3 times. In the younger-old age group, the risk increases by 2.8 times and in the older-old group by 2.1 times if the individual is not fully vaccinated. The results show that the decision of the younger-old population to get vaccinated led to a lesser risk of a lethal outcome. Evaluation of the effectiveness of the vaccination process in our sample showed results equivalent to the results of the studies done in other countries. A study done in Hungary showed greater effectiveness in the 65–74 years vs. 75–84 years age group for all vaccine types included in this research analysis (for BNT 1622b2 94.4% vs. 88.9%; Gam-COVID-Vac 96.5% vs. 95.1%; ChAdOx1 nCoV-19 97.8% vs. 96.5% and for BBIBP-CorV 87.1% vs. 82.2%) [12]. A retrospective study done in Colombia showed an age-dependent decrease in vaccine effectiveness. Prevention of hospitalization and death was highest in the 60–69-year age group, followed by the group aged 70–79 years, while in people aged 80 years and older vaccination showed the lowest effectiveness [32]. The several studies sampling the antibody titres after vaccination showed that older individuals have lower antibody levels, which indicate a decrease in B cell response [9,33,34].

Additional results from the regression model showed that every comorbidity individually has a noteworthy effect on the poor outcome. Conclusions regarding these results require further evidence and could be interpreted as encouragement for further research in this field.

All of the data collected, from the incidence of breakthrough infections through the severity type of COVID-19 to the final outcome, showed that vaccination has an unprecedented effect on saving the lives of older adults. An increase in age remains a true risk factor for COVID-19 because of the reduced capacity of organs to fight the disease and have a complete immune reaction to vaccination, causing breakthrough infections to be the most dangerous for older age groups. We cannot neglect the fact that this result can have an impact on facilitation of public health issues regarding COVID-19, through reducing hospital admissions, demand for intensive care units, the occurrence of disease complications, potential disability later on in life, as well as constant need for support and care.

The limitation of this study is its retrospective nature regarding the effect of vaccination. The results cannot be generalized because in other countries different types of vaccines were administered and achieved a larger coverage of the population immunized, making the term ‘herd immunity’ a relative ideal of health in each country.

## 5. Conclusions

The results of this retrospective, observational study in SARS-CoV-2-positive patients in Serbia showed that fully vaccinated individuals do have protective benefits from severe and critical forms and poor outcomes from COVID-19 compared to their unvaccinated control group. Age as an independent factor still contributes directly to poorer outcomes and severity. We advise prompt vaccination against SARS-CoV-2 as an efficient prevention method for maintaining good health in older patients. Further, more extensive, prospective, multicentric studies are encouraged to confirm and validate our results.

## Figures and Tables

**Table 1:** Comorbidity structure of participants according to vaccination status.

Variable			Total Population (n = 11,372)					Younger-Old (n = 7504)					Older-Old (n = 3868)		
Vaccination coverage (%)	48.4					51.3					46.2				
Vaccination status	Yes	No	df	Chi-square test	P	Yes	No	df	Chi-square test	P	Yes	No	df	Chi-square test	P
	1975 (17.4)	9397 (82.6)				1272 (17.0)	6232 (83.0)				703 (18.2)	3165 (81.8)			
Gender (m)	1021 (51.7)	4082 (43.4)	1	44.977	<0.001	632 (49.7)	2817 (45.2)	1	8.549	0.003	389 (55.3)	1265 (40.0)	1	55.490	<0.001
Survivors	1855 (93.9)	8193 (87.2)	1	71.997	<0.001	1232 (96.9)	5740 (92.1)	1	36.186	<0.001	623 (88.6)	2453 (77.5)	1	43.654	<0.001
Non-survivors	120 (6.1)	1204 (12.8)				40 (3.1)	492 (7.9)				80 (11.4)	712 (22.5)			
					Comorbidity										
Hypertension	641 (32.5)	3165 (33.7)	1	1.100	0.294	383 (30.1)	1864 (29.9)	1	0.020	0.887	258 (36.7)	1301 (41.1)	1	4.641	0.031
CVD	136 (6.9)	767 (8.2)	1	3.636	0.057	86 (6.8)	418 (6.7)	1	0.005	0.944	50 (7.1)	349 (11.0)	1	9.528	0.002
COPD	38 (1.9)	151 (1.6)	1	1.004	0.316	25 (2.0)	97 (1.6)	1	1.104	0.293	13 (1.8)	54 (1.7)	1	0.069	0.793
Diabetes mellitus	156 (7.9)	705 (7.5)	1	0.366	0.545	93 (7.3)	443 (7.1)	1	0.066	0.798	63 (9.0)	262 (8.3)	1	0.349	0.555
Obesity	31 (1.6)	134 (1.4)	1	0.235	0.627	16 (1.3)	107 (1.7)	1	1.381	0.240	15 (2.1)	27 (0.9)	1	8.784	0.003
Malignancy	14 (0.7)	110 (1.2)	1	3.226	0.072	10 (0.8)	72 (1.2)	1	1.332	0.248	4 (0.6)	38 (1.2)	1	2.137	0.144
Others	64 (3.2)	392 (4.2)	1	3.675	0.055	39 (3.1)	231 (3.7)	1	1.250	0.264	25 (3.6)	161 (5.1)	1	2.944	0.086

Data are shown as number (%); Abbreviations: CVD—cardiovascular disease; COPD—chronic obstructive pulmonary disease; Others: ischemic stroke, arrhythmias, renal insufficiency, psychiatric disorders, neurologic disabilities, valvular heart disease, peripheral vascular disease.

**Table 2:** Average age, unvaccinated status and comorbidities of the participants according to disease outcome.

Variable				Total Population (n = 11,372)					Younger-Old (n = 7504)					Older-Old (n = 3868)		
Outcome		S	NS	df	Test	*p*	S	NS	df	Test	*p*	S	NS	df	Test	*p*
Age	N	10,048	1324	11370	−26.366	<0.001 **	6972	532	7502	−5.877	0.451 **	3076	792	3866	−12.355	0.001 **
	Mean ± SD	72.5 ± 6.2	77.4 ± 7.4				69.0 ± 2.8	69.7 ± 2.8				80.4 ± 4.3	82.5 ± 4.7			
Unvaccinated		8193 (81.5)	1204 (90.9)	1	71.997	<0.001 *	5740 (82.3)	492 (92.5)	1	36.186	<0.001 *	2453 (79.7)	712 (89.9)	1	43.654	<0.001 *
						Comorbidity										
Hypertension		3196 (31.8)	610 (46.1)	1	106.911	<0.001 *	2035 (29.2)	212 (39.8)	1	26.783	<0.001 *	1161 (37.7)	398 (50.3)	1	40.959	<0.001 *
CVD		685 (6.8)	218 (16.5)	1	148.965	<0.001 *	435 (6.2)	69 (13.0)	1	35.740	<0.001 *	250 (8.1)	149 (18.8)	1	77.737	<0.001 *
COPD		141 (1.4)	48 (3.6)	1	35.344	<0.001 *	100 (1.4)	22 (4.1)	1	22.547	<0.001 *	41 (1.3)	26 (3.3)	1	14.069	<0.001 *
Diabetes mellitus		698 (6.9)	163 (12.3)	1	48.148	<0.001 *	461 (6.6)	75 (14.1)	1	41.758	<0.001 *	237 (7.7)	88 (11.1)	1	9.495	0.002 *
Obesity		140 (1.4)	25 (1.9)	1	2.004	0.157 *	112 (1.6)	11 (2.1)	1	0.652	0.419 *	28 (0.9)	14 (1.8)	1	4.311	0.038 *
Malignancy		90 (0.9)	34 (2.6)	1	30.334	<0.001 *	59 (0.8)	23 (4.3)	1	55.291	<0.001 *	31 (1.0)	11 (1.4)	1	0.852	0.356 *
Others		338 (3.4)	118 (8.9)	1	93.569	<0.001 *	218 (3.1)	52 (9.8)	1	62.973	<0.001 *	120 (3.9)	66 (8.3)	1	27.029	<0.001 *

Data are shown as number (%), mean ± SD as appropriate according to distribution. * Chi-square test, ** *t*-test. Abbreviations: CVD—cardiovascular disease; COPD—chronic obstructive pulmonary disease; Others: ischemic stroke, arrhythmias, renal insufficiency, psychiatric disorders, neurologic disabilities, valvular heart disease, peripheral vascular disease. S-Survivors; NS-Non-survivors.

**Table 3:** Binary logistic regression model for the prediction of lethal outcome of COVID-19 of total population.

Variable	B	S.E.	Wald	df	Sig.	Odds Ratio (OR)	95% CI for OR	
							Lower	Upper
Unvaccinated	0.854	0.102	69.649	1	<0.001	2.350	1.923	2.872
Age	0.095	0.004	491.702	1	<0.001	1.100	1.091	1.109
Hypertension	0.992	0.074	178.526	1	<0.001	2.697	2.332	3.120
COPD	1.712	0.185	85.762	1	<0.001	5.541	3.857	7.961
DM	1.296	0.108	144.793	1	<0.001	3.655	2.959	4.514
Obesity	1.250	0.230	29.462	1	<0.001	3.491	2.223	5.484
Malignancy	1.853	0.217	73.042	1	<0.001	6.382	4.172	9.762
Others	1.610	0.128	159.341	1	<0.001	5.004	3.897	6.426
Constant	−10.617	0.347	937.739	1	<0.001	0.000		

Abbreviations: COPD—chronic obstructive pulmonary disease; DM—diabetes mellitus; Others: ischemic stroke, arrhythmias, renal insufficiency, psychiatric disorders, neurologic disabilities, valvular heart disease, peripheral vascular disease.

**Table 4:** Binary logistic regression model for the prediction of lethal outcome of COVID-19 of Younger-old group.

Variable	B	S.E.	Wald	df	Sig.	Odds Ratio (OR)	95% CI for OR	
							Lower	Upper
Unvaccinated	1.037	0.170	37.302	1	<0.001	2.820	2.022	3.933
Age	0.085	0.016	27.069	1	<0.001	1.088	1.054	1.123
Hypertension	1.068	0.114	88.306	1	<0.001	2.910	2.329	3.635
COPD	1.840	0.254	52.533	1	<0.001	6.299	3.830	10.362
DM	1.522	0.153	98.912	1	<0.001	4.582	3.395	6.185
Obesity	1.014	0.329	9.470	1	<0.001	2.755	1.445	5.254
Malignancy	2.350	0.264	79.396	1	<0.001	10.482	6.252	17.575
Others	1.919	0.179	115.482	1	<0.001	6.816	4.803	9.673
Constant	−10.129	1.152	77.253	1	<0.001	0.000		

Abbreviations: COPD—chronic obstructive pulmonary disease; DM—diabetes mellitus; Others: ischemic stroke, arrhythmias, renal insufficiency, psychiatric disorders, neurologic disabilities, valvular heart disease, peripheral vascular disease.

**Table 5:** Binary logistic regression model for the prediction of lethal outcome of COVID-19 of Older-old group.

Variable	B	S.E.	Wald	df	Sig.	Odds Ratio (OR)	95% CI for OR	
							Lower	Upper
Unvaccinated	0.744	0.130	32.661	1	<0.001	2.105	1.631	2.717
Age	0.096	0.009	117.361	1	<0.001	1.101	1.082	1.120
Hypertension	0.926	0.099	88.108	1	<0.001	2.523	2.080	3.061
COPD	1.603	0.270	35.250	1	<0.001	4.967	2.926	8.431
DM	1.089	0.151	52.253	1	<0.001	2.970	2.211	3.990
Obesity	1.613	0.347	21.647	1	<0.001	5.016	2.543	9.893
Malignancy	1.113	0.367	9.183	1	<0.001	3.044	1.482	6.255
Others	1.324	0.177	55.847	1	<0.001	3.757	2.655	5.316
Constant	−10.469	0.736	202.326	1	<0.001	0.000		

Abbreviations: COPD—chronic obstructive pulmonary disease; DM—diabetes mellitus; Others: ischemic stroke, arrhythmias, renal insufficiency, psychiatric disorders, neurologic disabilities, valvular heart disease, peripheral vascular disease.

## Data Availability

The data presented in this study are available on request from the Vladimir Petrović Vladimir.
